# Genetic Prediction of the Phosphate‐to‐Glucose Ratio Mediates the Association Between CXCL5 and Vascular Dementia

**DOI:** 10.1002/brb3.70378

**Published:** 2025-03-26

**Authors:** Guifeng Zhuo, Wei Chen, Yanan Hu, Jinzhi Zhang, Xiaomin Zhu, Mingyang Su, Yulan Fu, Wu Lin

**Affiliations:** ^1^ Department of Neurology The First Affiliated Hospital of Guangxi University of Chinese Medicine Nanning China; ^2^ The First Clinical College of Medicine Guangxi University of Chinese Medicine Nanning China; ^3^ Ziwei comunity health care service center The Second Affiliated Hospital Cuhk‐Shenzhen Longgang District People's Hospital of Shenzhen Shenzhen China; ^4^ Scientific Laboratorial Centre Guangxi University of Chinese Medicine Nanning China

**Keywords:** causal relationship, CXCL5, Mendelian randomization, metabolite, phosphate‐to‐glucose ratio, vascular dementia

## Abstract

**Background and Objectives:**

A variety of observational studies suggest a possible connection between C‐X‐C Motif Chemokine Ligand 5 (CXCL5) and vascular dementia (VaD), though the exact causal relationship is still uncertain. This research aims to investigate the causal connection between CXCL5 and VaD risk through a Mendelian randomization (MR) method and to examine the phosphate‐to‐glucose ratio as a possible mediator.

**Methods:**

Using summary‐level data from genome‐wide association studies (GWAS), we conducted a two‐sample MR analysis to investigate the genetic prediction of CXCL5 and VaD. Horizontal pleiotropy, heterogeneity, and sensitivity analyses were also performed on the MR findings. Additionally, a two‐step MR was utilized to quantify the proportion of the effect of CXCL5 on VaD mediated by the phosphate‐to‐glucose ratio.

**Results:**

MR analysis identified that higher levels of CXCL5 (IVW: *p* = 0.022, OR = 1.265, 95% CI = 1.034–1.547) increase the risk of VaD. Tests for horizontal pleiotropy (*p* > 0.05), heterogeneity (*p* > 0.05), and sensitivity analyses supported these findings. There is insufficient robust evidence to suggest that genetic predispositions for VaD have any significant impact on CXCL5 (IVW: *p* = 0.254). The phosphate‐to‐glucose ratio accounted for 11.1% of increase in the risk of VaD associated with CXCL5 (95% CI = −12.3% to 34.5%).

**Conclusion:**

To conclude, our research confirms a causal link between CXCL5 and VaD and shows that the ratio of phosphate‐to‐glucose plays a mediating role in a segment of the risk effect of CXCL5 on VaD. However, most of the effects of CXCL5 on VaD are still not well understood. Additional studies are necessary to explore other potential mediators as risk factors. In clinical settings, individuals with abnormally elevated CXCL5 may need to be monitored for an increased risk of developing VaD.

## Abstract 2

Initial forward and reverse MR analyses were utilized to investigate the causal connection between CXCL5 and VaD. The beta value derived from the MR analysis (where CXCL5 is the exposure and VaD is the outcome) was designated as the total effect (beta‐all).

## 1 Introduction

Vascular dementia (VaD) is marked by worsening cognitive impairment and is widely recognized as the second most prevalent type of dementia in older adults, following Alzheimer's disease (Wolters and Ikram 2019). VaD results in ongoing and permanent declines in life quality, causing substantial medical and financial strain on families and society. However, due to the clinical heterogeneity and complexity of pathological types of VaD, the mechanisms underlying its onset are still not fully elucidated, and there remains a lack of safe and effective preventive and therapeutic measures.

The development of VaD is a multifaceted process that involves various pathological mechanisms, among which neuroinflammation plays a significant role (Kuang et al. 2021). Increasing evidence suggests that inflammatory factors are extensively involved in the pathological damage process of VaD (Li et al. 2021; Tian et al. 2022). However, the pathological progression of VaD can also lead to changes in the levels of inflammatory factors. The causal link between inflammatory markers and the risk of VaD onset has not yet been studied. Clarifying the causal relationships between specific inflammatory factors and VaD risk could facilitate the search for effective strategies to prevent and treat VaD. CXCL5, a member of the CXC chemokine subfamily, may play an important role in the progression of VaD. It has been reported that, following bilateral carotid artery stenosis to simulate a chronic cerebral ischemia state in mice, overexpression of CXCL5 in astrocytes exacerbated white matter damage and cognitive decline in these animals (Cao et al. 2023). However, studies related to CXCL5 and VaD are sparse, and their mutual causal effects have not been elucidated. Therefore, further research is necessary to clarify the direct link between CXCL5 and VaD.

Furthermore, the potential mediating pathways linking CXCL5 and VaD remain unexplored. Earlier research has indicated that metabolites undergo significant changes in both CXCL5 and VaD (Harusato et al. 2019; Meier et al. 2015; Qiang et al. 2024). Thus, metabolites may act as potential intermediaries between CXCL5 and VaD.

MR relies on genome‐wide association studies (GWAS) and is a reliable epidemiological research method. MR analysis uses single nucleotide polymorphisms (SNPs) as IVs to assess the causal impact of related exposures on outcomes, effectively minimizing the biases due to potential confounders, reverse causation, and the inherently small sample sizes typical of observational studies (Emdin et al. 2017). Thus, our aim is to establish if a causal connection exists between CXCL5 and VaD, and to evaluate the extent to which metabolites mediate the effect of CXCL5 on VaD.

## 2 Methods

### 2.1 Data Sources and Instrumental Variables

We sourced the GWAS summary statistics for CXCL5 from existing literature (Zhao et al. 2023). Summary data for 1091 metabolites and 309 metabolite ratios from a European cohort are accessible via the GWAS Catalog (https://www.ebi.ac.uk/gwas/), under accession numbers GCST90199621‐90201020 (Chen et al. 2023). The GWAS summary data for VaD were sourced from the Finnish database (https://www.finngen.fi/en), which includes data on 1256 VaD cases and 392,463 control subjects from the Finnish population. All summary data used in the article are from publicly available sources and can be downloaded for free. Each GWAS involved has received ethical approval from the respective institutions.

For more thorough results, we selected SNPs highly associated with inflammatory markers, metabolites, or VD (*p* < 1.0 × 10^−8^) as instrumental variables (IVs); If no SNPs met the genome‐wide significance threshold, those with significance levels below genome‐wide significance (*p* < 5 × 10^−6^ or *p* < 1 × 10^−5^) were considered as potential IVs. Linkage disequilibrium (LD) analysis based on genomic sample data was performed with parameters set tor^2^ < 0.001, kb = 10,000; the strength of the IVs was assessed by calculating the *F*‐statistic, with *F* > 10 indicating no weak instrument bias, and IVs with *F* < 10 were excluded. To satisfy the MR Independence hypothesis, we utilized the PhenoScanner V2 database to examine each IV and its proxy characteristics, subsequently excluding SNPs associated with confounding factors. Additionally, SNPs with a minor allele frequency (MAF) below 1% were excluded from the analysis.

### 2.2 Main Analysis

**FIGURE 1:**
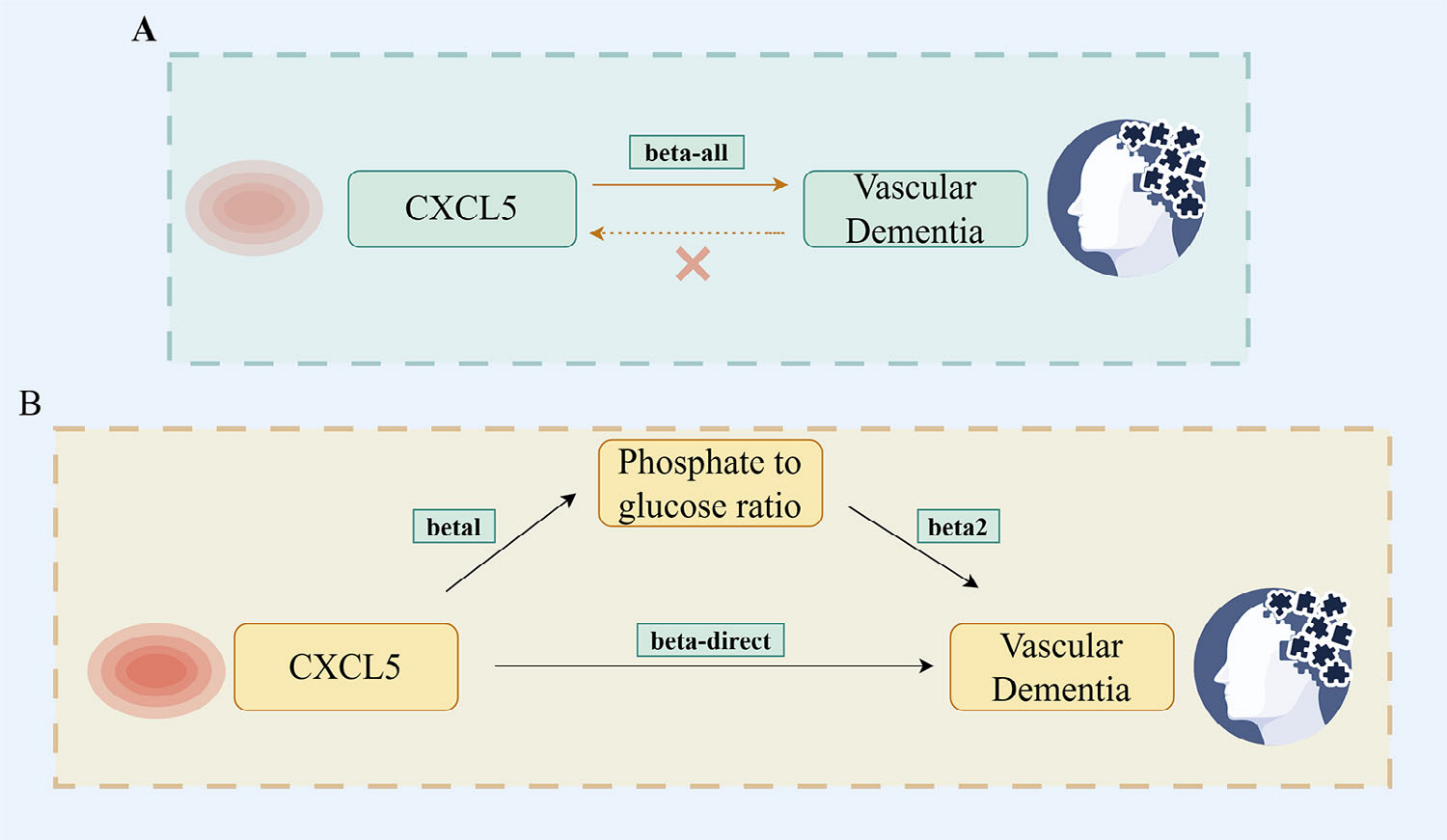
Diagrams illustrating associations examined in this study. (A) The total effect between CXCL5 and VaD. beta‐all is the total effect using genetically predicted CXCL5 as exposure and VaD as outcome. (B) The total effect was decomposed into (i) indirect effect using a two‐step approach (where beta1 is the total effect of CXCL5 on phosphate‐to‐glucose ratio, and beta2 is the effect of phosphate‐to‐glucose ratio on VaD) and the product method (beta1 × beta2) and (ii) direct effect (beta‐direct = beta‐all–beta1 × beta2). Proportion mediated was the indirect effect divided by the total effect. CXCL5, C‐X‐C Motif Chemokine Ligand 5; VaD, vascular dementia.

An overview of the analysis is shown in Figure 1. Initial forward and reverse MR analyses were utilized to investigate the causal connection between CXCL5 and VaD (Figure 1A). The beta value derived from the MR analysis (where CXCL5 is the exposure and VaD is the outcome) was designated as the total effect (beta‐all).

### 2.3 Preliminary Screening of Mediator Metabolites

To preliminarily identify suitable metabolites as mediator factors, the IVW was employed as the primary screening technique. An initial batch of MR analyses was conducted on the exposure factor CXCL5 and 1400 metabolic outcomes. Metabolites potentially having a causal relationship with CXCL5 were taken as exposure factors, and further batch MR analysis was performed with the outcome factor VaD. Ultimately, metabolites that were potentially related to both CXCL5 and VaD (*p* < 0.05) were identified and included as mediators for the next stage of mediation analysis.

### 2.4 Mediation Analysis

A two‐stage MR approach was used for mediation analysis to investigate whether initially screened metabolites mediated the causal pathway from CXCL5 to the VaD outcome (Figure 1B). Complete MR analyses were conducted on CXCL5 and the preliminarily screened metabolites, and between the metabolites and VaD, respectively. The overall effect is the sum of the indirect effect (through the mediator) and the direct effect (Carter et al. 2021). The total effect of CXCL5 on VaD (beta‐all) can be decomposed into CXCL5's direct effect on VaD (beta‐direct in Figure 1B) and the indirect effect mediated by the initially screened metabolites (beta1 × beta2 in Figure 1B). The proportion of the mediation effect is determined by dividing the indirect effect by the total effect. Moreover, the delta method is applied to compute the 95% confidence intervals (Yuan et al. 2022).

### 2.5 Statistical Analysis

In this research, two‐sample MR analyses were performed utilizing methods such as IVW, the weighted median method, MR–Egger regression, simple mode, and weighted mode to deduce causal connections (Bowden et al. 2015, 2016). IVW was employed as the primary method for estimating the overall effect size. IVW integrates the MR effect estimates of individual SNPs to produce a combined weighted estimate of potential causal effects, and is most reliable when there is no pleiotropy at the level of the IVs (Huang et al. 2021). MR analyses were conducted utilizing the R TwoSampleMR package.

Cochran's *Q* test was utilized to evaluate heterogeneity among SNPs (Greco et al. 2015). In cases where no significant heterogeneity was detected (*p* > 0.05), a fixed‐effects model was applied; otherwise, a random‐effects model was used. The MR–Egger intercept (intercept *p* value < 0.05) was used as an indicator to assess horizontal pleiotropy (Burgess and Thompson 2017). Additionally, sensitivity analysis was carried out using the “leave‐one‐out” method to assess the influence of individual SNPs on the causal relationship. All statistical analyses were conducted with R software version 4.3.0.

## 3 Results

### 3.1 Causal Effect of C‐X‐C Motif Chemokine Ligand 5 (CXCL5) on VaD

According to the criteria for selecting IVs, 27 SNPs associated with CXCL5 and 16 SNPs related to VaD were identified, each having an *F*‐statistic exceeding 10, suggesting that the study is less likely to be biased by weak instruments. The study primarily relied on the IVW, setting *p* < 0.05 as the threshold for conducting bidirectional two‐sample MR analysis between CXCL5 and VaD.

**FIGURE 2:**
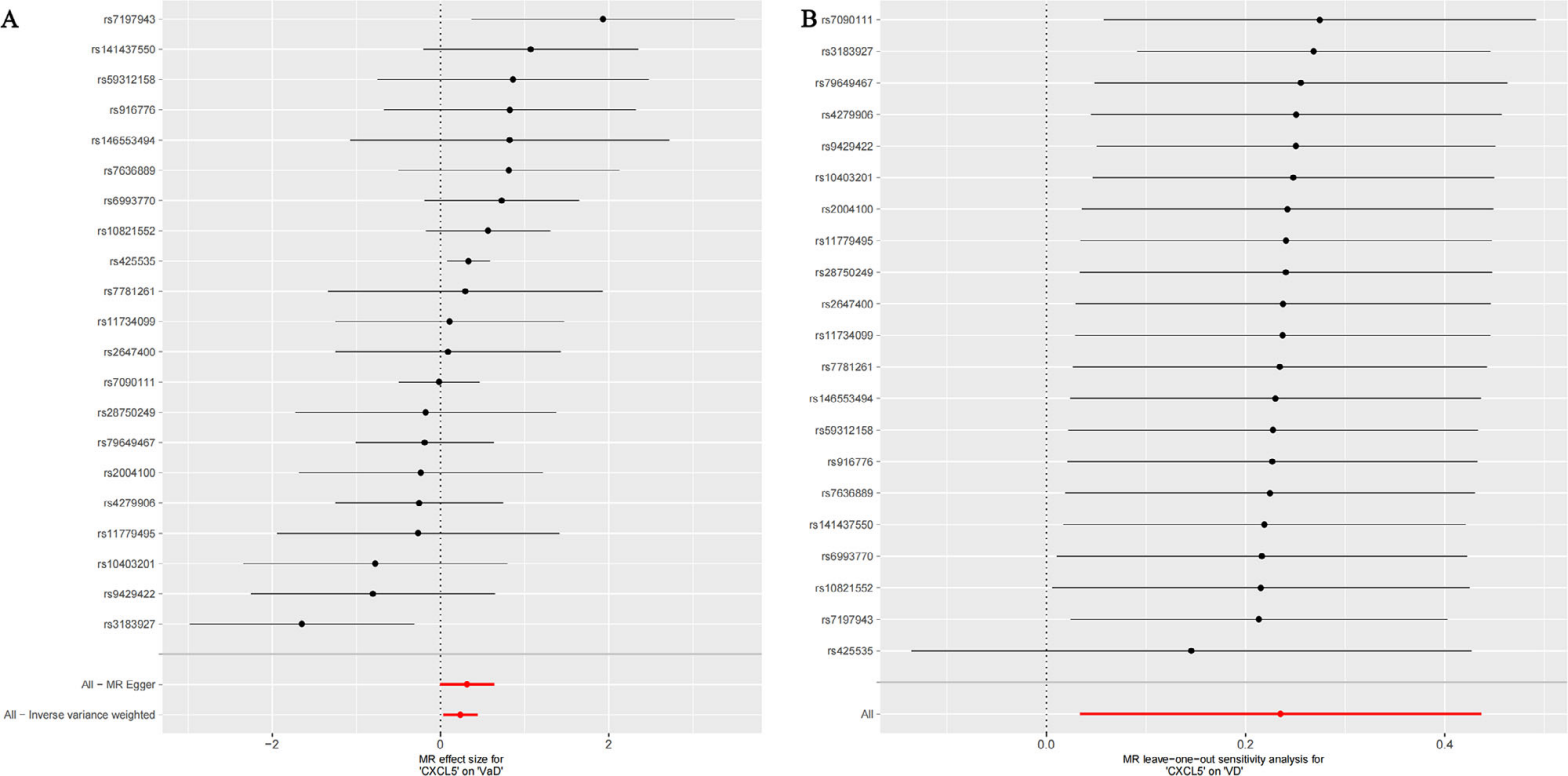
Forest plots displaying the effect of CXCL5 on VaD using MR analysis (A) and leave‐one‐out analysis of the causal relationship between CXCL5 and VaD (B). CXCL5, C‐X‐C Motif Chemokine Ligand 5; VaD, vascular dementia.

The results of the forward MR analysis (Table 1, Figure 2A) indicate that an increase in the expression level of CXCL5 (IVW: *p* = 0.022, OR = 1.265, 95% CI = 1.034–1.547) is linked to a heightened risk of VaD. Moreover, the MR–Egger intercept test (Table 2) provided no indication of horizontal pleiotropy. Heterogeneity tests indicated no significant heterogeneity among the selected IVs. A leave‐one‐out sensitivity analysis was carried out, in which each SNP was excluded one at a time. The causal effects of the remaining SNPs were then compared to those from the complete MR analysis to identify whether a single IV was responsible for driving the causal link. The sensitivity analysis demonstrated that the MR results were robust (Figure 2B). The outcomes of the reverse MR analysis, where VaD served as the exposure dataset, showed no causal relationship with CXCL5 (IVW: *p* = 0.254).

**TABLE 1:** The findings from the Mendelian randomization analysis examining the causality of CXCL5 with phosphate‐to‐glucose ratio and VaD.

Exposure	Outcome	MR–Egger		Weighted median		Inverse variance weighted		Simple mode		Weighted mode	
		OR (95%CI)	*p* value	OR (95%CI)	*p* value	OR (95%CI)	*p* value	OR (95%CI)	*p* value	OR (95%CI)	*p* value
CXCL5	VaD	1.369 (0.994–1.885)	0.070	1.375 (1.088–1.738)	0.008	1.265 (1.034–1.547)	0.022	0.983 (0.576–1.679)	0.951	1.319 (1.045–1.666)	0.030
VaD	CXCL5	1.017 (0.967–1.069)	0.522	1.016 (0.982–1.053)	0.360	1.015 (0.989–1.042)	0.254	1.021 (0.971–1.075)	0.430	1.015 (0.978–1.053)	0.440
CXCL5	Phosphate‐to‐glucose ratio	0.914 (0.823–1.015)	0.109	0.913 (0.836–0.997)	0.043	0.907 (0.846–0.971)	0.005	0.841 (0.692–1.021)	0.094	0.904 (0.825–0.992)	0.045
Phosphate‐to‐glucose ratio	VaD	0.776 (0.563–1.071)	0.141	0.786 (0.565–1.093)	0.153	0.767 (0.624–0.942)	0.011	0.852 (0.528–1.373)	0.518	0.798 (0.592–1.075)	0.154

**TABLE 2:** An examination of the pleiotropic effects and heterogeneity observed in the relationship of CXCL5 with phosphate‐to‐glucose ratio and VaD.

Exposure	Outcome	MR–Egger intercept		MR–Egger			Inverse variance weighted		
		Egger intercept	*p* value	*Q*	*Q*_*df*	Q*_p*val	*Q*	*Q_df*	*Q_p*val
CXCL5	VaD	−0.014	0.536	25.754	19.000	0.137	26.291	20.000	0.156
CXCL5	Phosphate‐to‐glucose ratio	−0.001	0.841	16.188	20.000	0.705	16.229	21.000	0.757
Phosphate‐to‐glucose ratio	VaD	−0.002	0.922	10.800	18.000	0.903	10.810	19.000	0.930

### 3.2 Preliminary Screening Results for Mediator Metabolites

The initial batch MR analysis of CXCL5 with 1400 metabolites and metabolite ratios identified 66 metabolites potentially having a causal relationship with CXCL5 (Table 3). Further preliminary batch MR analysis of these 66 metabolites with VaD revealed three metabolites and metabolite ratios potentially related to VaD: X‐23648 levels (IVW: *p* = 0.047), phosphate‐to‐glucose ratio (IVW: *p* = 0.011), and phosphate to citrate ratio (IVW: *p* = 0.024). The metabolite ratio with the smallest *p* value and potential causal relationship with VaD, phosphate‐to‐glucose ratio, was selected as the mediator for the next step of mediation analysis.

**TABLE 3:** Initial analysis of CXCL5 and 1400 metabolites and their ratios.

Immfator name	Dxwd id	*p* value (inverse variance weighted)
CXCL5	GCST90200161	0.038
CXCL5	GCST90200165	0.009
CXCL5	GCST90200372	0.011
CXCL5	GCST90200382	0.022
CXCL5	GCST90200332	0.026
CXCL5	GCST90200393	0.008
CXCL5	GCST90200396	0.005
CXCL5	GCST90200625	0.007
CXCL5	GCST90200600	0.022
CXCL5	GCST90200610	0.022
CXCL5	GCST90200964	0.034
CXCL5	GCST90200967	0.005
CXCL5	GCST90200968	0.004
CXCL5	GCST90200427	0.044
CXCL5	GCST90200261	0.017
CXCL5	GCST90200270	0.018
CXCL5	GCST90200297	0.019
CXCL5	GCST90199844	0.029
CXCL5	GCST90199875	0.037
CXCL5	GCST90200081	0.015
CXCL5	GCST90199986	0.010
CXCL5	GCST90199992	0.015
CXCL5	GCST90201011	0.018
CXCL5	GCST90201012	0.031
CXCL5	GCST90200979	0.004
CXCL5	GCST90199625	0.038
CXCL5	GCST90199637	0.040
CXCL5	GCST90199638	0.017
CXCL5	GCST90199640	0.007
CXCL5	GCST90199899	0.018
CXCL5	GCST90199903	0.002
CXCL5	GCST90199905	0.014
CXCL5	GCST90199906	0.009
CXCL5	GCST90199910	0.027
CXCL5	GCST90200036	0.044
CXCL5	GCST90200046	0.033
CXCL5	GCST90200052	0.022
CXCL5	GCST90200058	0.020
CXCL5	GCST90200063	0.046
CXCL5	GCST90200578	0.010
CXCL5	GCST90200709	0.010
CXCL5	GCST90200767	0.005
CXCL5	GCST90200764	0.015
CXCL5	GCST90200772	0.002
CXCL5	GCST90200103	0.046
CXCL5	GCST90200725	0.024
CXCL5	GCST90200733	0.018
CXCL5	GCST90200740	0.027
CXCL5	GCST90199788	0.031
CXCL5	GCST90199793	0.015
CXCL5	GCST90199805	0.025
CXCL5	GCST90199773	0.017
CXCL5	GCST90199775	0.033
CXCL5	GCST90200531	0.014
CXCL5	GCST90200505	0.045
CXCL5	GCST90200676	0.035
CXCL5	GCST90199736	0.018
CXCL5	GCST90200494	0.041
CXCL5	GCST90200904	0.031
CXCL5	GCST90200943	0.023
CXCL5	GCST90200952	0.012
CXCL5	GCST90200861	0.006
CXCL5	GCST90200918	0.015
CXCL5	GCST90200835	0.029
CXCL5	GCST90200785	0.003
CXCL5	GCST90200794	0.031

### 3.3 Causal Effect of CXCL5 on the Metabolite Ratio Phosphate‐to‐Glucose Ratio

**FIGURE 3:**
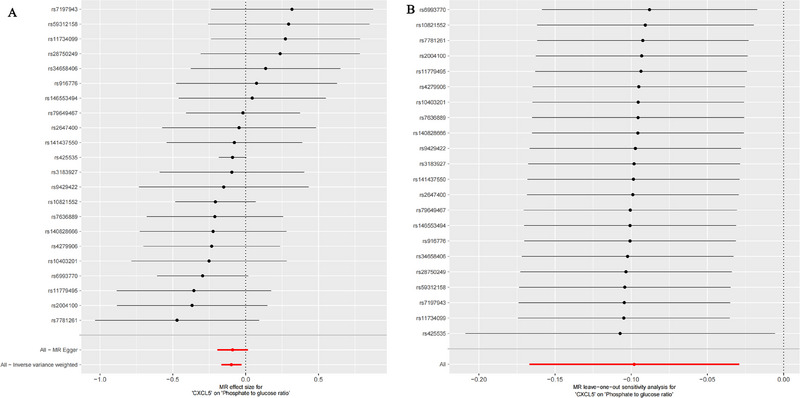
Forest plots displaying the effect of CXCL5 on phosphate‐to‐glucose ratio using MR analysis (A) and leave‐one‐out analysis of the causal relationship between CXCL5 and phosphate‐to‐glucose ratio (B). CXCL5, C‐X‐C Motif Chemokine Ligand 5; MR, Mendelian randomization.

MR analysis results (Table 1, Figure 3A) show that an increase in the expression level of CXCL5 (IVW: *p* = 0.005, OR = 0.907, 95% CI = 0.846–0.971) is associated with a decrease in the phosphate‐to‐glucose ratio level. Additionally, as indicated in Table 2, the MR–Egger intercept test revealed no evidence of horizontal pleiotropy, and there was no significant heterogeneity among the selected IVs, indicating robust MR analysis results (Figure 3B).

### 3.4 Causal Effect of Phosphate‐to‐Glucose Ratio on VaD

**FIGURE 4:**
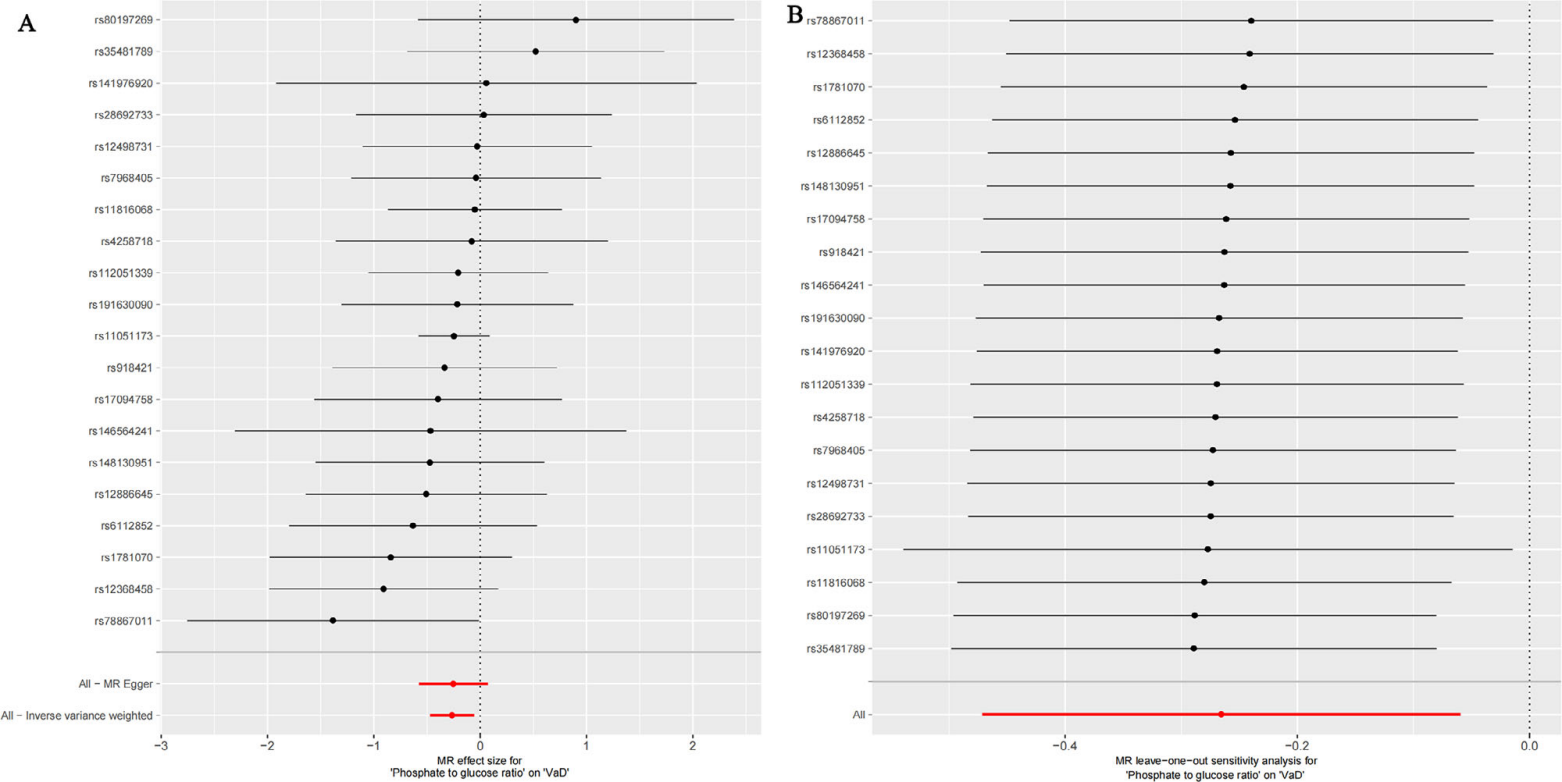
Forest plots displaying the effect of phosphate‐to‐glucose ratio on VaD using MR analysis (A) and leave‐one‐out analysis of the causal relationship between phosphate‐to‐glucose ratio and VaD (B). MR, Mendelian randomization; VaD, vascular dementia.

The MR analysis results (Table 1, Figure 4A) indicate that an increase in the phosphate‐to‐glucose ratio (IVW: *p* = 0.011, OR = 0.767, 95% CI = 0.624–0.942) is linked to a decreased risk of developing VaD. Additionally, the MR–Egger intercept test in Table 2 did not indicate horizontal pleiotropy, and there was no significant heterogeneity among the chosen IVs, ensuring robust MR analysis results (Figure 4B).

### 3.5 Mediation Proportion of the Phosphate‐to‐Glucose Ratio Between CXCL5 and VaD

We analyzed the role of the phosphate‐to‐glucose ratio as a mediator in the pathway from CXCL5 to VaD. We found that a reduction in the phosphate‐to‐glucose ratio, associated with increased CXCL5, is linked to an increased risk of VaD. As shown in Table 2, our study indicates that the total effect of CXCL5 on VaD is 0.235, with the indirect effect mediated by the phosphate‐to‐glucose ratio being 0.026. This represents 11.1% of the increase in risk for VaD related to CXCL5 (proportional mediation: 11.1%; 95% CI = −12.3% to 34.5%).

## 4 Discussion

The fundamental pathophysiology of VaD is based on sustained low cerebral perfusion, leading to a deficiency in essential nutrients such as oxygen, glucose, and amino acids, which triggers central nervous system tissue damage or even necrosis, promoting the onset and progression of neuroinflammation (Han et al. 2020). Indeed, inflammatory states are present in the hippocampal tissue of VaD patients, characterized by elevated levels of pro‐inflammatory cytokines such as tumor necrosis factor‐alpha (TNF‐α), interleukin‐1β (IL‐1β), transforming growth factor‐beta (TGF‐β), and inducible nitric oxide synthase (iNOS) (Belkhelfa et al. 2018). This neuroinflammation, in turn, damages neurons, leading to cell apoptosis and necroptosis, further impairing cognitive and learning functions (Ni et al. 2019), thus creating a vicious cycle that promotes the progressive development of VaD. Thus, neuroinflammation is both a driver and a product of the pathological progression of VaD. Inflammatory factors are direct participants in neuroinflammation; however, whether they act as a cause or consequence in the disease process of VaD remains poorly understood. Therefore, this study seeks to elucidate the causal role of the inflammatory marker CXCL5 in the risk of developing VaD and to evaluate the extent to which metabolites mediate the effect of CXCL5 on VaD.

In this study, we employed genetic tools to comprehensively examine the causal relationship between CXCL5 and VaD for the first time, demonstrating this relationship through existing GWAS data and assessing whether it is mediated by the phosphate‐to‐glucose ratio. By integrating the results from both forward and reverse Mendelian randomization (MR), we discovered that genetically predicted elevated levels of CXCL5 are linked to a 26% higher risk of VaD per one standard deviation increase, with 11.1% of this effect mediated through the phosphate‐to‐glucose ratio. Additionally, there is no evidence to support any causal impact of genetically predicted VaD on CXCL5. This suggests that CXCL5 may serve as an early predictive factor for the development of VaD, but not as a biomarker for its prognosis.

CXCL5, also referred to as the CXC chemokine subfamily, epithelial neutrophil‐activating peptide‐78 (ENA‐78) is produced by cells such as neuroglial and epithelial cells (Sepuru et al. 2014). The expression of CXCL5 is markedly elevated following central nervous system damage, where it participates in neuroinflammatory responses and neuronal apoptosis (Wang et al. 2016). As far as we know, this is the initial research to examine the causal link between CXCL5 and VaD employing the MR approach, also demonstrating that the phosphate‐to‐glucose ratio is one of the mediators involved. Our results align with previous research findings. For example, Cao et al. (2023) found that astrocyte overexpression of CXCL5 in a chronic cerebral hypoperfusion mouse model exacerbated white matter damage and cognitive decline. Similarly, Xiao et al. (2022) conducted a cohort study that found elevated levels of CXCL5 in the circulation of elderly populations with small vessel disease, suggesting its potential utility in diagnosing and monitoring vascular cognitive impairments. However, these studies have their limitations. First, the study by Cao et al. suffers from a small sample size and uses non‐human subjects. Second, the study by Guanxi Xiao et al. is observational in design, making it prone to confounding variables and reverse causation. Unlike the findings of Guanxi Xiao and others, our MR analysis does not support using CXCL5 as a biomarker for the prognosis of VaD.

Metabolites, which are the intermediate or final products of metabolic processes, are small molecules whose concentrations can be affected by multiple factors, such as genetics, lifestyle, gut microbiota, and disease conditions (Bar et al. 2020, Lee and Hase 2014, Pietzner et al. 2021). They also play a role in affecting disease risk and may act as potential targets for therapeutic interventions (Wishart 2016). Metabolites and their ratios are crucial in the health and disease states of the brain. In a longitudinal cohort study, Huang et al. (2023) found that metabolites such as glucose and cholesteryl ester in small High‐Density Lipoprotein (HDL) particles were associated with the risk of VaD. Additionally, increased levels of butyrates and the butyrate ratio have been reported to exert a protective effect on the neurons in VaD mice (Liu et al. 2015). Exploring the causal influence of metabolites and their ratios in the development of VaD could offer actionable targets for intervention and prevention strategies. However, current studies exploring the causal relationships between metabolites, their ratios, and VaD are scarce. Our study is the first to identify a potential simultaneous causal relationship involving CXCL5, VaD, the phosphate‐to‐glucose ratio, X‐23648 levels, and the phosphate‐to‐citrate ratio. Notably, the phosphate‐to‐glucose ratio emerges as a significant indicator of alterations in glucose metabolism, exhibiting the smallest *p* value and suggesting a causal link with VaD. Given that glucose metabolism is the primary energy source for brain cells, disruptions in this process are recognized as early indicators of dementia (Zhao et al. 2011). Subsequent investigations revealed that rats with VaD frequently exhibited pathological alterations, including glucose metabolism disorders and mitochondrial dysfunction at the cellular level (Wu et al. 2023). Consequently, this study conducted further MR analysis of the phosphate‐to‐glucose ratio. The findings indicate that an elevated phosphate‐to‐glucose ratio mitigates the risk of VaD and may serve as an intermediary mechanism through which CXCL5 influences VaD susceptibility. This insight could help identify metabolic biomarkers for the prediction and early diagnosis of VaD.

The primary advantage of this MR study lies in its ability to mitigate the influence of confounding factors. Genetic variations in CXCL5, the phosphate‐to‐glucose ratio, and VaD were derived from the largest available genome‐wide association study meta‐analysis, thereby ensuring the robustness of the MR analysis. Simultaneously, Cochran's *Q* test and MR–Egger intercept analysis demonstrated the absence of horizontal pleiotropy and heterogeneity within this study, thereby corroborating the reliability of the study's findings. Nonetheless, the study is not without its limitations. Initially, the investigation was performed using a European cohort, hence the applicability of our findings to populations outside of Europe remains to be examined. Additionally, this study was based on summary‐level data as opposed to individual‐level data. As a result, we could not delve into the causal relationships between different subtypes of VaD, or within specific demographic groups such as men and women. Furthermore, our research shows that the genetic prediction of VaD mediated by the phosphate‐to‐glucose ratio is 11.1%, which is comparatively modest. Consequently, additional studies are required to identify further mediators. It is noteworthy that this study necessitated a greater number of genetic variants IVs for sensitivity analysis and the detection of horizontal pleiotropy. Consequently, some of the SNPs employed in the analysis did not satisfy the significance threshold established by traditional GWAS (*p *< 1 × 10^−8^). Therefore, to substantiate the findings of this study, future research should be undertaken to further validate the causal relationship between CXCL5, the phosphate‐to‐glucose ratio, and VaD. Our results also imply that CXCL5 may elevate the risk of VaD via X‐23648 levels and the phosphate to citrate ratio, but these possibilities require further verification through tests for pleiotropy, heterogeneity, and additional metabolomic experiments.

## 5 Conclusions

In conclusion, our research confirms a causal link between CXCL5 and VaD and demonstrates that the phosphate‐to‐glucose ratio mediates part of CXCL5's risk impact on VaD. However, the majority of CXCL5's effects on VaD remain uncertain. Additional studies are necessary to investigate other potential mediators as risk factors. In clinical settings, individuals with elevated levels of CXCL5 may need to be concurrently monitored for their risk of developing VaD.

## Author Contributions

**Guifeng Zhuo**: writing–original draft, writing–review and editing. **Wei Chen**: writing–original draft, writing–review and editing, supervision. **Yanan Hu**: writing–original draft, investigation. **Jinzhi Zhang**: data curation, investigation. **Xiaomin Zhu**: formal analysis. **Mingyang Su**: data curation. **Yulan Fu**: validation, visualization. **Lin Wu**: conceptualization, writing–original draft, writing–review and editing.

## Ethics Statement

This study is based on a published database and does not require ethical approval.

## Conflicts of Interest

The authors declare no conflict of interest.

### Peer Review

The peer review history for this article is available at https://publons.com/publon/10.1002/brb3.70378.

## Data Availability

Publicly available datasets were analyzed in this study.
